# Unraveling the identity of FoxP3+ regulatory T cells in Granulomatosis with Polyangiitis patients

**DOI:** 10.1038/s41598-019-44636-y

**Published:** 2019-06-04

**Authors:** Tom D. Y. Reijnders, Coen A. Stegeman, M. G. Huitema, Abraham Rutgers, Peter Heeringa, Wayel H. Abdulahad

**Affiliations:** 1 Department of Rheumatology and Clinical Immunology, University of Groningen, University Medical Center Groningen, Groningen, The Netherlands; 2 Department of Internal Medicine, Division of Nephrology, University of Groningen, University Medical Center Groningen, Groningen, The Netherlands; 3 Department of Pathology and Medical Biology, University of Groningen, University Medical Center Groningen, Groningen, The Netherlands

**Keywords:** Autoimmunity, Interleukins, CD4-positive T cells, Nephritis, Vasculitis syndromes

## Abstract

Human CD4^+^*FoxP3*^+^T-cells are heterogeneous in function and include not only suppressive cells (Tregs), but also effector cells that transiently express *FoxP3* upon activation. Previous studies in Granulomatosis with Polyangiitis (GPA-)patients have demonstrated an increase in *FoxP3*^+^T-cells with impaired suppressive capacity and an increase in Th17 cells. We hypothesized that the increase in *FoxP3*^+^T-cells results from an increase in non-suppressive effector-like cells. The frequency of circulating CD4^+^*FoxP3*^+^T-cell subsets were determined by flow cytometry in 46 GPA-patients in remission and 22 matched healthy controls (HCs). Expression levels of *FoxP3* and CD45RO were used to distinguish between CD45RO^−^ *FoxP3*^low^ resting Tregs (rTreg), CD45RO^+^*FoxP3*^high^ activated Tregs (aTreg) and CD45RO^+^*FoxP3*^low^ proinflammatory non-suppressive T-cells (nonTreg). Intracellular expression of IFNγ, IL-17, and IL-21 was compared within these subsets. We found a significant increase in the frequency of nonTreg cells in GPA-patients as compared with HCs. Importantly, within the nonTreg subset, antineutrophil cytoplasmic autoantibody (ANCA-)positive patients demonstrated a significantly higher percentage of IL-17+ and IL-21+ cells when compared with ANCA-negative patients and HCs. Moreover, expanded nonTregs from ANCA-positive patients induced excessive proliferation of responder cells *in vitro* and exhibited higher IL-21 production. Production of IL-17 and IL-21 in non-suppressive *FoxP3*^+^T-cells may point toward a pathogenic role in ANCA formation.

## Introduction

Granulomatosis with Polyangiitis (GPA) is a necrotizing granulomatous autoimmune small-vessel vasculitis distinguished by circulating antineutrophil cytoplasmic autoantibodies (ANCA) that predominantly target proteinase 3 (PR3)^1^. ANCA-induced neutrophil-mediated tissue damage has classically been deemed the primary pathogenic mechanism^2^. However, accumulating evidence points towards an essential role for T cells in disease expression and progression: effector T cells are found in biopsies of inflamed renal and granulomatous tissue^3,4^; ANCAs are class-switched autoantibodies and therefore necessitate T cell help^5^; increased serum markers of T cell activation correlate with disease activity^6,7^; and peripheral blood homeostasis of CD4+ T cells is disturbed with the persistent expansion of effector memory cells during remission^8,9^.

Autoimmunity in GPA implies a critical defect in the tolerance to self-antigens. Human regulatory T cells (Tregs) are key mediators of peripheral tolerance that can actively inhibit inflammation and suppress effector T cell function and proliferation. Tregs are characterized by the presence of *FoxP3*, a transcription factor that is critical to their development and function^10^. Our group previously demonstrated that GPA-patients in disease remission exhibit a marked increase in circulating T cells expressing *FoxP3*^11^. Yet this increase failed to confer enhanced suppression, as these cells were functionally defective^11^, a finding since corroborated by others^12,13^.

*FoxP3* has been considered a master regulator of Treg development and function: unique to the Treg lineage^14–16^. However, effector T cells may transiently express *FoxP3* upon activation, without acquiring suppressor function^17^. Distinguishing suppressive and non-suppressive *FoxP3*+ T cells has proved challenging, due to the ambiguity of conventional markers^10^. Miyara *et al*. classified *FoxP3*+ T cells by the level of *FoxP3* expression and whether these cells possessed a naïve or memory phenotype and found three distinct populations: CD45RO− *FoxP3*^low^ resting Tregs (rTreg), CD45RO+ *FoxP3*^high^ activated Tregs (aTreg) and CD45RO+ *FoxP3*^low^ proinflammatory cytokine secreting non-suppressive T cells (nonTreg)^18^. Furthermore, even suppressive Tregs may acquire the ability to produce effector cytokines (such as IL-17) upon activation in the context of a proinflammatory milieu^19–21^.

In addition to *FoxP3*+ T cells, our group reported increased frequencies of circulating T cells producing IL-17^22^, as well as IL-21^23^, in GPA-patients.

Based on these observations, we hypothesized that the overabundance of functionally defective *FoxP3*+ T cells results from an expansion of effector-like cells that readily produce proinflammatory cytokines despite expression of *FoxP3*. Elucidating the functional and phenotypical characteristics of *FoxP3*+ T cells in GPA may form a next step in the search for novel monitoring tools and possible directed therapies.

## Results

### The increase in *FoxP3*+ T cells in GPA is predominantly due to an expansion of nonTreg cells

**Figure 1:**
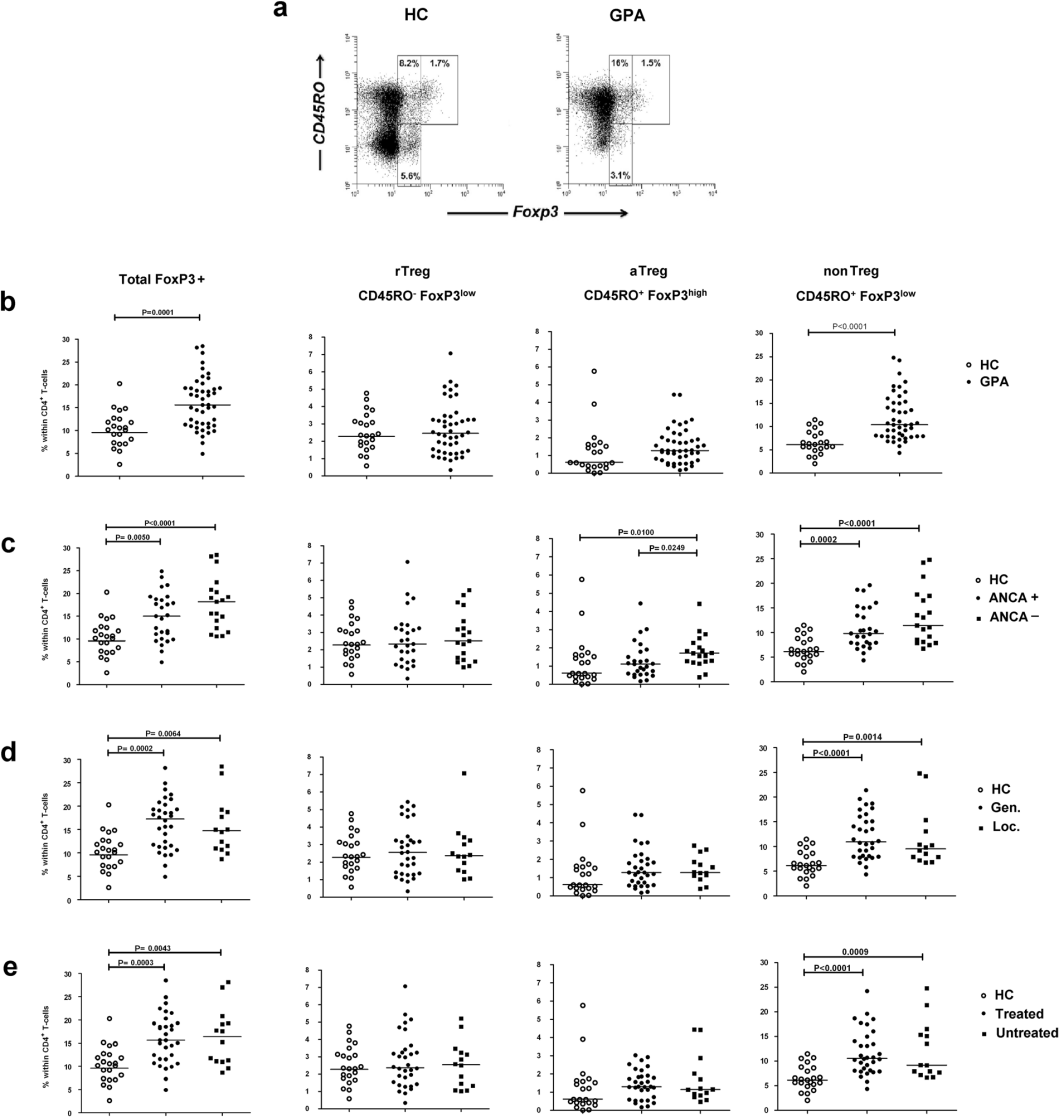
Percentages of circulating Treg subpopulations in GPA-patients and HCs. (**a**) Representative flow cytometry plots of CD45RO and FoxP3 expression on gated CD4+ T cells in freshly isolated PBMCs obtained from one HC (left) and one GPA-patient (right) for determining the frequencies of CD45RO− *FoxP3*^low^ resting Tregs (rTreg), CD45RO+ *FoxP3*^high^ activated Tregs (aTreg) and CD45RO+ *FoxP3*^low^ proinflammatory cytokine secreting non-suppressive T cells (nonTreg). The percentages of circulating *FoxP3*+ CD4+ T cells and the percentages of the Treg subsets within CD4+ T cells were compared between: (**b**) HCs and GPA-patients; (**c**) HCs, ANCA-positive (ANCA+; ANCA titer higher than 1:20 at time of blood sampling) and ANCA-negative (ANCA−) GPA-patients; (**d**) HCs and GPA-patients with generalized (Gen.) or localized (Loc.) disease; (**e**) HCs and treated and untreated GPA-patients. Horizontal lines in the scatterplots represent the median. *P*-values were calculated using the nonparametric Mann-Whitney U-test.

We first analyzed the percentages of total *FoxP3*+ cells within CD4+ T cells in peripheral blood of GPA-patients in remission and matched healthy controls (HCs). In line with our previously published data^11^, the frequency of circulating *FoxP3*+ T cells in GPA-patients was significantly increased when compared with HCs (Fig. 1b). No differences were found between patients who were currently ANCA-negative or ANCA-positive (Fig. 1c), patients with localized or generalized disease (Fig. 1d), or untreated patients and those receiving maintenance therapy (Fig. 1e).

To evaluate the identity of the *FoxP3*+ T cells, we subdivided these cells according to the three populations described by Miyara *et al*.^18^ (Fig. 1a). Strikingly, we found that the increase in *FoxP3*+ T cells in GPA-patients is largely caused by an increase in the nonTreg subset (Fig. 1b). When compared with HCs, the median proportion of nonTreg cells within CD4+ T cells was about twice as large in ANCA-positive patients (p = 0.0002) and ANCA-negative patients (p < 0.0001; Fig. 1c). The percentages of the rTreg subset were comparable between ANCA-positive patients, ANCA-negative patients and HCs (Fig. 1c).

Whereas ANCA-positive and ANCA-negative patients did not differ in nonTregs and rTregs, ANCA-negative patients showed a significantly expanded population of aTregs when compared with both ANCA-positive patients and HCs (Fig. 1c). These data indicate that, in GPA-patients, an expansion of aTregs is accompanied by an absence of circulating ANCAs. As with total *FoxP3*+ T cells, no differences were found when comparing the three Treg subsets between patients based on disease localization or treatment status at the time of blood sampling (Fig. 1d,e).

### NonTreg cells from ANCA-positive patients produce more proinflammatory cytokines

**Figure 2:**
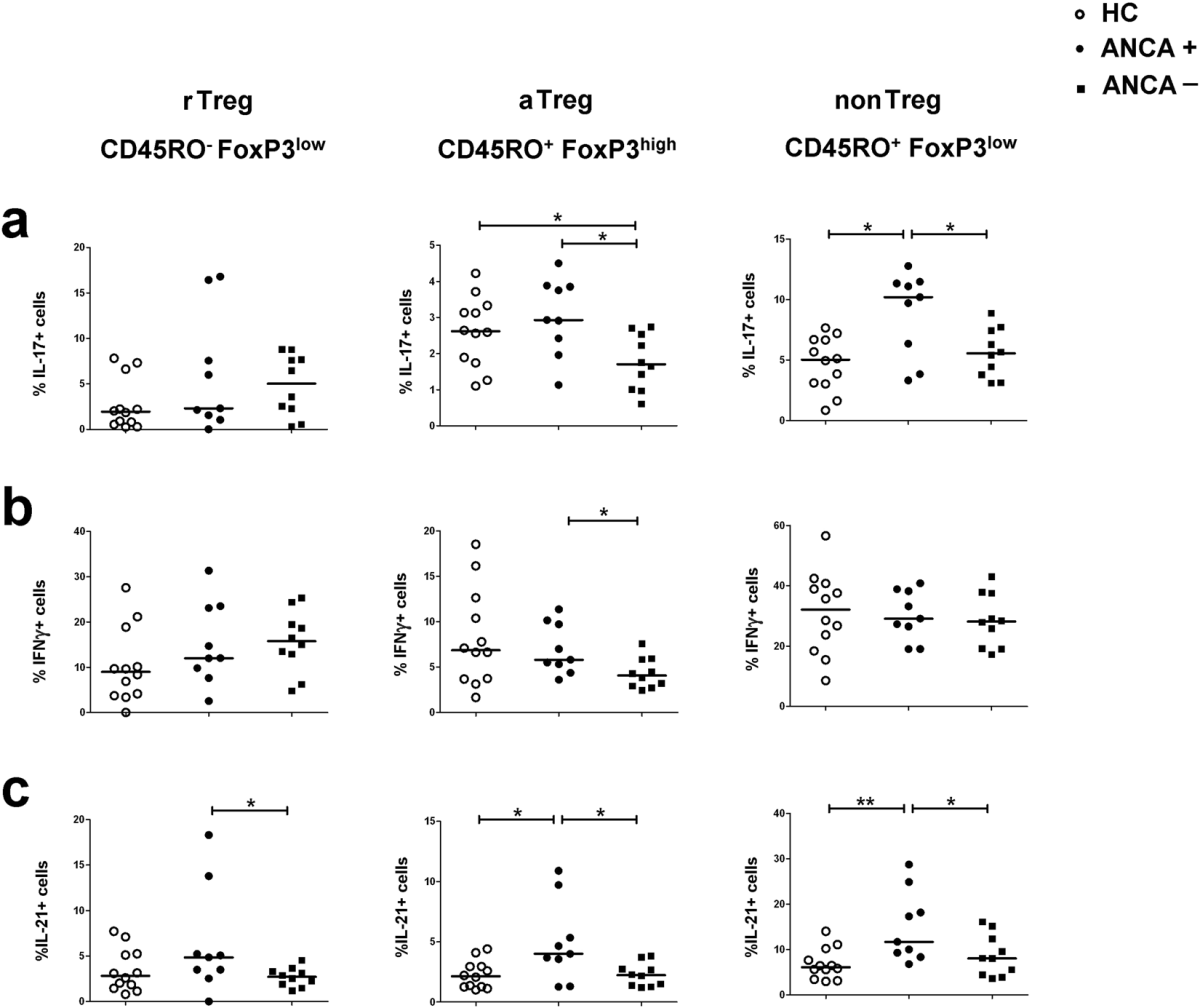
Percentages of cytokine secreting Treg cells in GPA-patients and HCs. Proportion of rTreg, aTreg and nonTreg cells producing IL-17 (**a**), IFNγ (**b**) or IL-21 (**c**) in ANCA-positive (ANCA+; ANCA titer higher than 1:20 at time of blood sampling) GPA-patients (n = 9), ANCA-negative (ANCA−) GPA-patients (n = 10), and HCs (n = 12) Horizontal lines in the scatterplots represent the median. *P*-values were calculated using the nonparametric Mann-Whitney U-test.

Next, we determined the intracellular expression of the proinflammatory cytokines IL-17, IFNγ and IL-21 in the three *FoxP3*+ T cell subsets after *in vitro* activation of PBMCs by PMA and calcium ionophore. Representative flow cytometry plots can be found in Supplementary Fig. S1. As expected, the nonTreg subset contained the highest frequencies of proinflammatory cytokine producing cells (Fig. 2). Within the nonTreg subset, ANCA-positive patients produced significantly more IL-17 and IL-21 (Fig. 2a,c) – but not IFNγ (Fig. 2b) – than both ANCA-negative patients and HCs, underscoring a link between these two cytokines and circulating ANCAs. In similar fashion, aTregs from ANCA-positive patients produced more IFNγ, IL-17 and IL-21 than aTregs from ANCA-negative patients (Fig. 2a–c). The rTreg subset displayed no differences in cytokine expression between any of the groups.

### *In vitro* expanded nonTreg cells from ANCA-positive patients induce responder T cell proliferation and produce more IL-21

**Figure 3:**
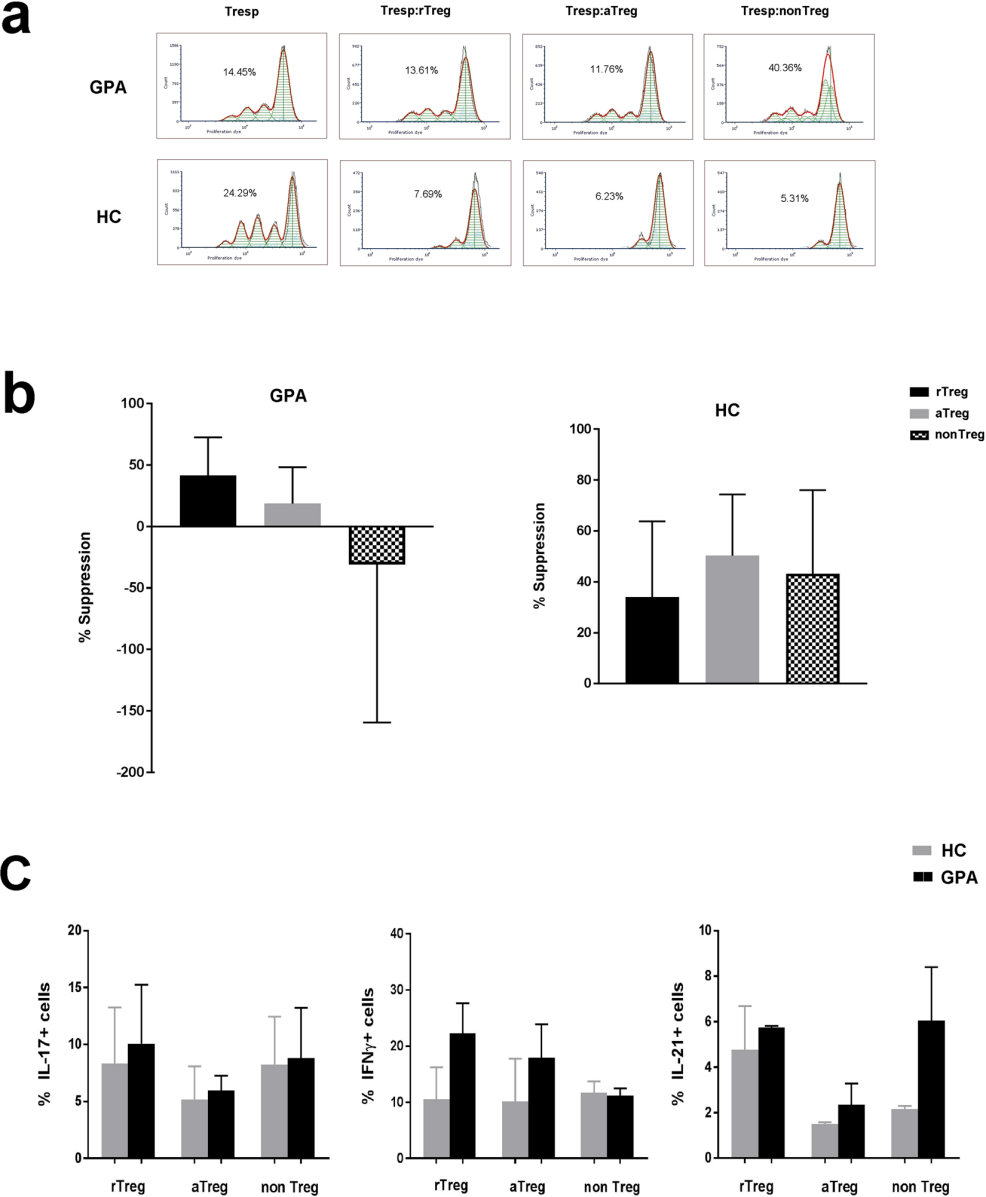
The suppressive capacity and cytokine pattern of *in vitro* expanded Treg subsets from ANCA-positive patients and HCs. Responder T (Tresp) cells were labeled with proliferation dye eFluor670 and stimulated to proliferate with anti-CD3/CD28 beads in the presence or absence of each *in vitro* expanded autologous Treg subset (rTreg, a Treg and nonTreg) separately in a 1:1 ratio. After 3 days, proliferation dye dilution was determined by flow cytometry and used to calculate suppression. (**a**) Representative Tresp cell proliferation dye dilution histograms for the different co-cultures. Each peak represents a successive generation of divided Tresp cells. (**b**) Bar charts comparing the percentage of suppression in each co-culture experiment between GPA-patients (n = 3) and HCs (n = 3). The bar represents the median and the whiskers represent the range. (**c**) Frequency of IL-17, IFNγ, and IL-21 production by *in vitro* expanded Treg subsets (rTreg, aTreg and nonTreg) from GPA-patients (n = 2) and HCs (n = 2).

We next assessed the suppressive capacities of the three Treg subsets in ANCA-positive GPA-patients and compared the results with those of matched HCs. We isolated and expanded the three Treg subsets *in vitro* and subsequently co-cultured them with autologous responder T cells (Tresp; CD4+  CD25−). We calculated suppression for each subset using Tresp proliferation. Although the limited sample size prevents drawing definitive conclusions, the results are intriguing nonetheless (Fig. 3). As expected, the overall suppressive capacity was reduced in GPA-patients (Fig. 3b). The nonTreg subset in HCs actually appeared to regain the ability to suppress after *in vitro* expansion. In stark contrast, the nonTreg subset in GPA-patients induced excessive proliferation of Tresp cells. Increased IL-21 production in these cells further underlines their effector-like phenotype (Fig. 3c). These preliminary data implicate the nonTreg subset in the exacerbation of cell-mediated inflammation in GPA-patients.

## Discussion

Our results clarify the identity of the expanded *FoxP*3+ T cells in GPA-patients. The increase in *FoxP3*+ T cells can be attributed to an expansion of the cytokine producing effector non-suppressive Treg subset (nonTregs). In ANCA-positive patients, these cells produced more of the proinflammatory cytokines IL-17 and IL-21. Moreover, *in vitro* expanded nonTregs from ANCA-positive patients caused excessive proliferation of T effector cells and exhibited higher IL-21 production. Combined with their defective suppressor function, these observations indicate a pathogenic role for *FoxP3*+ T cells in ANCA formation.

Our data reveal the identity of *FoxP3*+ T cells in GPA, but questions remain regarding their origins. Miyara and colleagues^24^ consider the non-suppressive subset to be derived from naïve CD4+ cells that transiently upregulate *FoxP3* upon activation. An alternative explanation stems from the plasticity between Tregs and Th17 cells, two cell lineages presumed to originate from the same precursor^25^. IL-17+ *FoxP3*+ cells, which express the Th17 transcription factor RORγt, can be generated in the presence Th17-inducing cytokines such as IL-1β, IL-6, IL-21 and IL-23^19–21^. *FoxP3* antagonizes RORγt function^26^ and high expression therefore appears protective against an IL-17 producing phenotype^20^. In contrast, the alternatively spliced isoform lacking exon 2 (*FoxP3Δ2)* fails to effect this inhibition^26^. Free *et al*. demonstrated an overrepresentation of *FoxP3Δ2* in patients with ANCA-associated vasculitis^13^. Collectively, we speculate that the IL-17+ *FoxP3*+ cells found here partially originate from cells with increased susceptibility to Th17 plasticity – expressing low levels of *FoxP3* or predominantly expressing *FoxP3Δ2*. IL-17+ *FoxP3*+ T cells are present in inflammatory lesions in autoimmune diseases such as rheumatoid arthritis^27^, psoriasis^28^ and inflammatory bowel disease^29^. As of yet, no subset of Tregs has been described that produce IL-21. Therefore, it is likely that the IL-21+ *FoxP3*+ cells found here represent activated effector memory cells.

Our group and others previously demonstrated skewing towards a Th17 phenotype in GPA^22,30,31^ and an expanded T effector memory population that persists during disease remission^8,9^. Our current finding is in line with these observations and suggests that the expanded non-suppressive *FoxP3*+ T cell subset with a memory phenotype may be an important source of proinflammatory cytokines. IL-17 is a critical player in autoimmunity, partly through its effects on the innate immune system. Secretion of IL-17 drives the production of various chemokines that recruit neutrophils^32^ and stimulates macrophages to produce IL-1β and TNF-α^33^, two proinflammatory cytokines that prime neutrophils^34^. In GPA, primed neutrophils translocate PR3 to their cell membrane. Subsequent interaction of PR3 with PR3-ANCA causes neutrophils to degranulate, followed by a sequence of proinflammatory events culminating in necrotizing vasculitis^34^. Thus, IL-17 likely plays a key initiating role in the pathogenesis of GPA.

In addition to IL-17, *FoxP3*^*+*^ T cells from ANCA-positive patients also produced more IL-21, which is in accordance with our previous data^23^. IL-21 induces pathogenic effector cells (mainly Th17) and causes them to expand^35,36^. Additionally, it may impair homeostasis and function of *bona fide* Tregs via suppression of *FoxP3* and decreased availability of IL-2^36,37^, or by making T effector cells refractory to suppression^38^. Importantly, IL-21 stimulates (auto)antibody production, antibody class switching and it synergizes with B cell activating factor to promote plasma cell generation^39,40^. Accordingly, IL-21 significantly enhances *in vitro* PR3-ANCA generation in ANCA-positive GPA-patients^23^.

Furthermore, we found that ANCA- negative patients have higher frequencies of aTregs than ANCA-positive patients. It is conceivable that the expansion of this population results in suppression of ANCA formation, as Tregs may directly inhibit autoantibody production by B cells in autoimmune disease^41^. Conversely, the elevated levels of IL-21 in ANCA positive patients may culminate in enhanced autoantibody production, as well as reduced levels of aTregs. While it is impossible to infer cause and effect from these data alone, it seems apparent that IL-21 plays a critical role in ANCA production and disturbances in Treg homeostasis in GPA.

The results of the suppression assay suggest that the expanded population of nonTregs in GPA-patients not only fail to suppress, but may even enhance effector T cell proliferation. The need for *in vitro* expansion of the small number of cells obtained from sorting may limit translation to the *in vivo* situation. The small sample size is another obvious limitation of this assay. Despite these caveats, the experimental design here is unprecedented and the results provide valuable insight into the consequential functional differences dividing the Treg subsets of GPA-patients and healthy controls.

Our data demonstrate that the overabundance of circulating *FoxP3*+ T cells in GPA-patients predominantly results from an expansion of the non-suppressive nonTregs. Within this subset, ANCA-positive patients produced significantly more of the proinflammatory cytokines IL-17 and IL-21, pointing to their role in disease pathogenesis. Further research is needed to elucidate the origins of these cells – in particular whether the IL-17+ *FoxP3*+ cells represent inherent and/or microenvironmental susceptibility to Th17 plasticity – and to confirm their pathogenicity in GPA. Understanding the role of *FoxP3*+ T cells in GPA may pave the way for novel monitoring tools and possible therapeutic targets.

## Methods

### Study population

46 consecutive patients with GPA (Table 1) and 22 age- and sex-matched healthy controls (15 males, 7 females, mean age 52 years, range 20–79 years) were enrolled in this study. The main clinical and laboratory data of the patients are summarized in Table 1. The diagnosis of GPA was established according to the definitions of the Chapel Hill Consensus Conference^42^ and fulfilled the classification criteria of the American College of Rheumatology^43^ for GPA. Only patients without clinical signs and symptoms of active vasculitis and considered to be in complete remission, as indicated by the Birmingham vasculitis activity score (BVAS)^44^, were included in this study. According to these criteria, all GPA-patients were in remission at the time of sampling. Of the 46 patients, 32 were considered *generalized*-GPA that included renal involvement, and 14 patients were considered *localized*-GPA which is confined to the upper and/or lower respiratory tract. None of the patients and controls experienced an infection at the time of sampling. 32 patients were receiving maintenance therapy at time of sampling, as specified in Table 1. All patients and healthy individuals provided informed consent and the study was approved by the local medical ethics committee (METc2012/151). The patient selection criteria and definitions described in this section are similar to those in our previous work on T cells in GPA to ensure consistency and facilitate comparisons between studies^11,23^.

**Table 1:** Clinical and laboratory characteristics of the GPA-patients.

Characteristic	Patients (n = 46)
Male/female	31/15
Age, mean (range) years	54 (20–78)
Localized/generalized GPA	14/32
Positive/negative for ANCA^†^	27/19
Receiving/not receiving treatment	32/14
Azathioprine	11
25 mg/day	1
50 mg/day	4
100 mg/day	3
150 mg/day	3
Prednisolone and azathioprine	9
Prednisolone and mycophenolate mofetil	5
Prednisolone and cyclophosphamide	4
Cyclosporine	1
Cotrimoxazole	2
Disease duration, mean (range) months	78 (5–266)

*ANCA* Anti neutrophil cytoplasmic antibody, *GPA* Granulomatosis with polyangiitis.

^†^ANCA titer higher than 1:20 at time of blood sampling. All included patients were ANCA-positive at initial diagnosis.

### Serum ANCA titer

ANCA titers were measured by indirect immunofluorescence (IIF) on ethanol-fixed human granulocytes according to the standard procedure as previously described^45^. ANCA titers higher than 1:20 were considered positive. All included patients were ANCA-positive at initial diagnosis with specificity for proteinase-3 (PR3-ANCA).

### Antibodies

The following conjugated anti-human antibodies were used in flow cytometry: Pacific Blue conjugated (PB) anti-CD3 (clone UCHT1), allophycocyanin (APC) or peridin-chlorophyll (PerCP) conjugated anti-CD4 (clone SK3), fluorescein isothiocyanate (FITC) or phycoerythrin-Cyanin7 (PE-Cy7) conjugated anti-CD45RO (clone UCHL1), and PerCP conjugated anti-CD8 (clone SK1). All antibodies were purchased from Becton-Dickinson (Amsterdam, The Netherlands). PE or APC conjugated anti-human *FoxP3* (clone PCH101), Alexa Fluor700 conjugated anti-IFNγ (clone 4S.B3), Alexa Fluor488 conjugated anti-IL-17 (clone eBio64Dec17), and PE conjugated anti-IL21 (clone eBio3A3-N2) were obtained from eBioscience (San Diego, CA, USA). Isotype matched control antibodies of irrelevant specificity were purchased from Becton-Dickinson and eBioscience.

### Isolation of peripheral blood mononuclear cells (PBMC)

Isolation of PBMCs was performed as described previously^11,23^. Briefly, peripheral blood was obtained by venipuncture in heparinized tubes, and PBMC were immediately isolated by density-gradient centrifugation on Lymphoprep (Axis-Shield PoC AS, Oslo, Norway). Cells were washed two times in phosphate-buffered saline pH 7.2 (PBS), and resuspended at 1 × 10^7^ cells/mL in RPMI 1640 (Cambrex Bio Science, Verviers, Belgium) supplemented with 5% human pool serum and 50 μg/ml gentamycin (Gibco, Scotland, UK).

### Flow cytometry staining to determine the frequency of circulating Treg cell subsets

*Foxp3* staining was performed according to the manufacturer’s instructions (*Foxp3-*staining kit, eBioscience, ITK diagnostic BV, The Netherlands) as described previously^11,23^. Briefly, freshly isolated PBMC (1 × 10^6^ cells in 100 μL) were immediately incubated with appropriate concentrations of FITC-anti-CD45RO, and PerCP-anti-CD4 for 30 minutes at 4 °C in the dark, washed with cold PBS followed by fixation and permeabilization in Fix/Perm buffer (eBioscience) for 45 minutes at 4 °C. Subsequently, cells were washed twice with cold 1X Permeabilization buffer (eBioscience). To block non-specific binding, normal rat serum was added for 10 minutes, followed by the addition of PE-conjugated rat anti-human-*FoxP3*. After incubation for 30 minutes at 4 °C, the cell suspension was washed twice with cold 1X Permeabilization buffer and four-color staining was immediately analyzed on FACS-Calibur (Becton & Dickinson). For all flow cytometry analyses, data were collected for 10^5^ lymphocytes, gated by forward and side scatter, and plotted using Kaluza software package (Beckman Coulter, USA). CD4^+^ T cells were gated and the expression levels of *FoxP3* and CD45RO were used for distinction between rTregs (CD45RO− *FoxP3*^low^), aTregs (CD45RO+ *FoxP3*^high^) and nonTregs (CD45RO+ *FoxP3*^low^) as shown in Fig. 2A and described by Miyara and colleagues^18^.

### Stimulation assay and immunofluorescent intracellular staining for cytokines

Immediately after isolation, 1 × 10^6^ PBMCs were resuspended in 400 μl RPMI1640 (Cambrex Bio Science, Verviers, Belgium), supplemented with 50 μg/ml gentamycin (Gibco, Paisley, Scotland, UK), and aliquoted into 5 ml polypropylene tubes (BD Biosciences, Amsterdam, The Netherlands). To determine the frequency of cytokine expressing Treg subsets, cells were stimulated for 16 h with 8 nM phorbol myristate acetate (PMA; Sigma-Aldrich, Steinheim, Germany) and 0.4 nM calcium ionophore (Ca-Io; Sigma-Aldrich) in the presence of 3 μM Brefeldin A. Brefeldin A was used to block intracellular transport mechanisms, thereby leading to an accumulation of cytokines in the cell. As a negative control, one sample remained without stimulation. Culture tubes were incubated at 37 °C, 5% CO_2_.

After stimulation, cells were washed in wash buffer (PBS, 5% fetal bovine serum (FBS)) and stained with PerCP-anti-CD8 (clone SK1, BD Biosciences, Amsterdam, The Netherlands) and APC-anti-CD3 (clone UCHT1, BD Biosciences), for 15 minutes at room temperature. Next, cells were washed, fixed, and permeabilized using the *Foxp3-*staining kit from eBioscience as mention before. After permeabilization, cells were stained intracellularly by adding APC-conjugated rat anti-human-*FoxP3*, Alexa Fluor 700-anti-IFNγ, Alexa Fluor 488-anti-IL-17, and PE-anti-IL-21 and incubated for 30 minutes at 4 °C. The cell suspension was washed and analyzed directly on LSR-II (Becton & Dickinson). Data were collected for 10^5^ lymphocytes and plotted using Kaluza software package (Beckman Coulter, USA). Treg cell subsets were gated separately and the frequency of IFNγ or IL-17 or IL-21 secreting cells were measured within each subset.

### Cell sorting and *in vitro* expansion of Treg cell subsets

Freshly isolated PBMCs from 3 ANCA-positive GPA-patients and 3 age- and sex-matched HCs were stained with anti-CD4-eF450, anti-CD25-PE and anti-CD45RO-FITC, and sorted on a MoFlo-Astrios (Beckman Coulter) according to Miyara’s classification^18^ into the following 4 populations: rTreg (CD4^+^CD25^+^CD45RO^−^), aTreg (CD4^+^CD25^High^CD45RO^+^), non-Treg (CD4^+^CD25^Low^CD45RO^−^) and responder T cells (Tresp; CD4^+^CD25^−^) (Supplementary Fig. S2). Tresp cells were frozen in liquid nitrogen until use. The 3 other Tregs fractions were expanded *in vitro* for 2 weeks using anti-CD3/CD28 Dynabeads (Thermo Fisher Scientific) and 200IU of IL-2 (Peprotech, Rocky Hill, USA) in RPMI1640 (Lonza, Breda, The Netherlands) supplemented with 10% human pooled serum (Lonza) and 60 µg/ml gentamycin sulfate (Lonza). Part of the 3 expanded Treg subsets was used in suppression assays (as described below) to determine their suppressive capacity, and another part was frozen and used later to assess cytokine production (as described above).

### Suppression Assay

Tresp cells were thawed, labeled with proliferation dye eFluor670 (eBioscience), stimulated with anti-CD3/CD28 Dynabeads and cultured (2 × 10^4^ cells/well) in a round-bottomed 96-well plate in the presence or absence of each autologous Treg subset (rTreg, aTreg and nonTreg) separately at a 1:1 ratio. After 3 days of culture, cells were harvested, washed and stained with Zombie UV Fixable Viability dye (Biolegend) and analyzed on an LSR-II (Becton & Dickinson). Dead cells were excluded and Tresp cell proliferation was determined by following eFluor670 dilution using FCS-express^TM^ 6 software (De *Novo Software*, Glendale, CA). The percentage suppression of proliferation was calculated as follows:

$$ \% {\rm{Suppression}}=\frac{ \% {\rm{proliferation}}\,{\rm{Tresp}}\,{\rm{alone}}- \% {\rm{proliferation}}\,{\rm{Tresp}}\,{\rm{in}}\,{\rm{coculture}}\,{\rm{with}}\,{\rm{Treg}}}{ \% {\rm{proliferation}}\,{\rm{Tresp}}\,{\rm{alone}}}\times 100 \% $$

### Statistical analysis

Data are presented as median unless stated otherwise. Comparison of median values between GPA-patients and healthy controls was assessed using nonparametric Mann-Whitney U-test, and differences were considered statistically significant at two-sided *P*-values less than 0.05.

### Ethics approval and consent to participate

All patients and healthy controls provided informed consent before participating in the study. The study was approved by the Medical Ethics Committee of the University of Groningen/University Medical Center Groningen, The Netherlands (METc2012/151). All procedures were in accordance with the Declaration of Helsinki.

## Supplementary information

Supplementary Information

## Data Availability

The datasets used and/or analyzed during the current study are available from the corresponding author on reasonable request.
