# Supplementary material for: Unraveling the identity of FoxP3+ regulatory T cells in Granulomatosis with Polyangiitis patients
Source: Sci Rep. 2019 Jun 4;9:8273. doi: 10.1038/s41598-019-44636-y (PMC6547679; doi:10.1038/s41598-019-44636-y)
Supplement: Supplementary file 1 — Supplementary Information [file 41598_2019_44636_MOESM1_ESM.pdf]

Unraveling the identity of FoxP3+ regulatory T cells in Granulomatosis with Polyangiitis patients

Supplementary Information

Tom D.Y. Reijnders[1], Coen A. Stegeman[2], M.G. Huitema[1], Abraham Rutgers[1], Peter Heeringa[3], Wayel H. Abdulahad[1,3]*

[1]Department of Rheumatology and Clinical Immunology, University of Groningen, University Medical Center Groningen, Groningen, the Netherlands.
[2]Department of Internal Medicine, Division of Nephrology, University of Groningen, University Medical Center Groningen, Groningen, the Netherlands.
[3]Department of Pathology and Medical Biology, University of Groningen, University Medical Center Groningen, Groningen, the Netherlands.

*** Corresponding author:**
Wayel H. Abdulahad, PhD.
Rheumatology and Clinical Immunology, AA21,
University of Groningen, University Medical Center Groningen
Hanzeplein 1, 9713 GZ, Groningen, The Netherlands
e-mail: w.abdulahad@umcg.nl
Tel:+31(0)50 36 19270
Fax: +31(0)50361 930

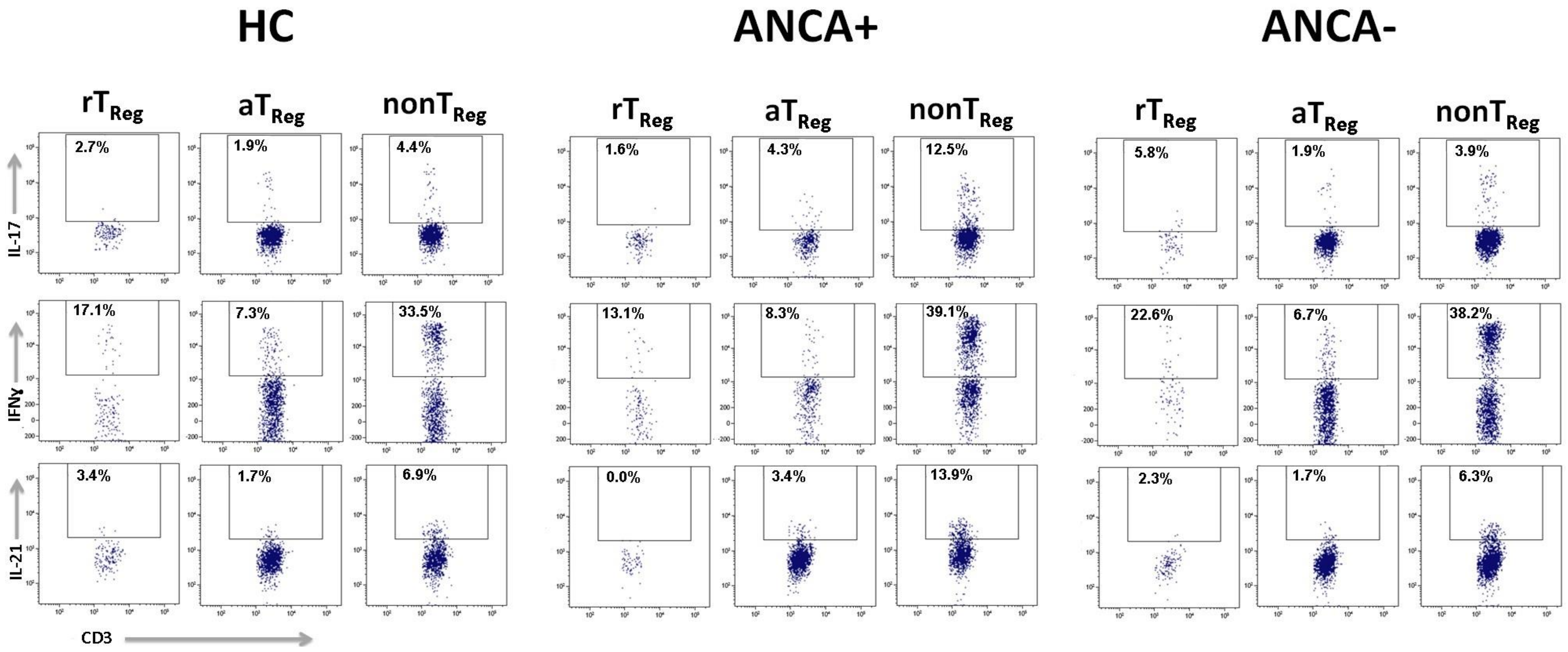

Supplementary Figure S1. Representative flow cytometry plots for the intracellular expression of IL-17, IFNγ and IL-21 in rTreg, aTreg and nonTreg cells in HCs, ANCA-positive (ANCA+) and ANCA-negative (ANCA-) GPA-patients.

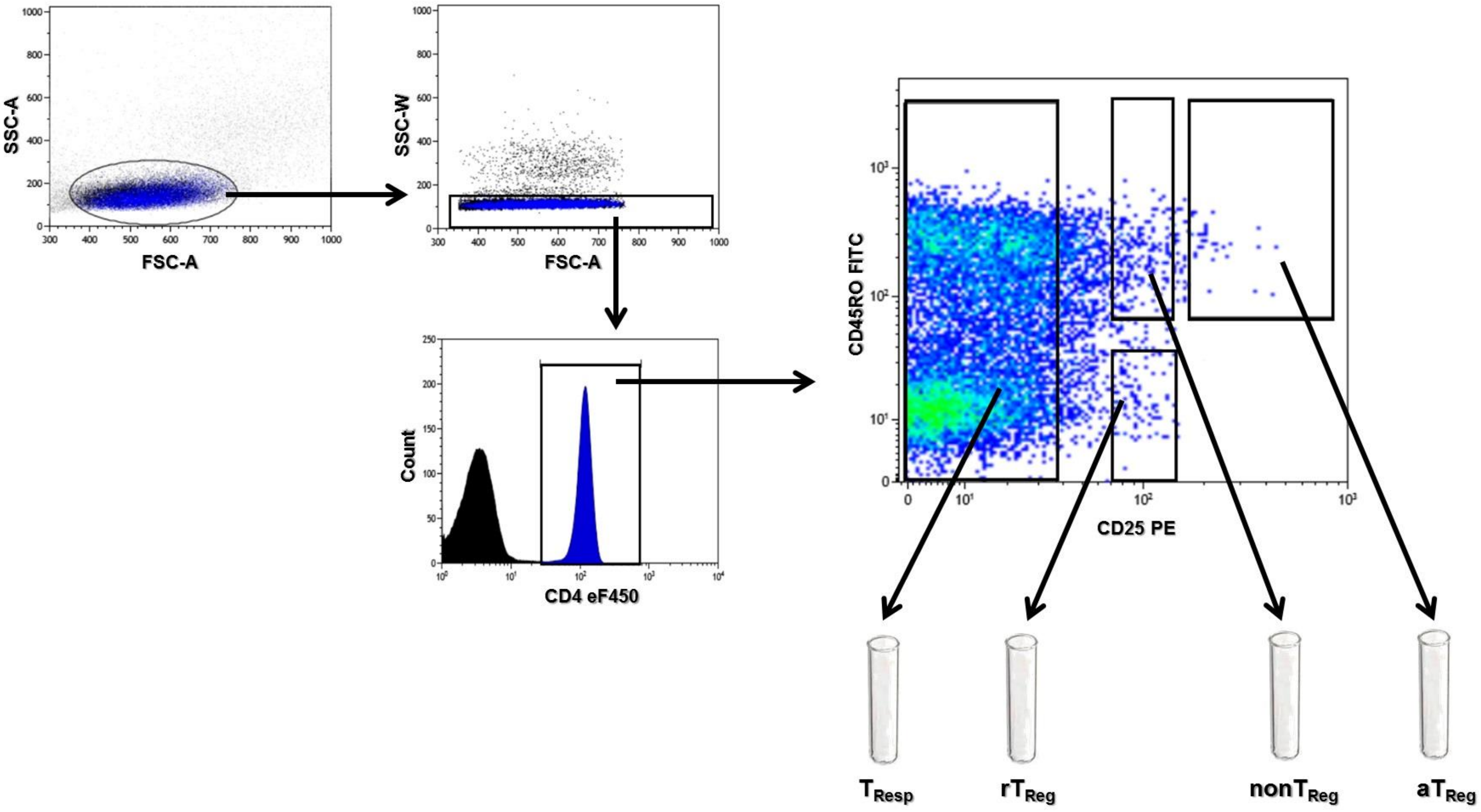

Supplementary Figure S2. Sorting strategy. PBMCs were stained with fluorochrome-conjugated antibodies against CD4, CD25 and CD45RO. Singlet lymphocytes were gated based on forward and side scatter (FSC and SSC) characteristics and CD4 expression and then divided into four populations: responder T cells (Tresp; $CD4^{+}CD25^{-}$), rTreg ($CD4^{+}CD25^{+}CD45RO^{-}$), nonTreg ($CD4^{+}CD25^{Low}CD45RO^{-}$) and aTreg ($CD4^{+}CD25^{High}CD45RO^{+}$).
